# Unilateral adrenal hyperplasia is a usual cause of primary hyperaldosteronism. Results from a Swedish screening study

**DOI:** 10.1186/1472-6823-12-17

**Published:** 2012-09-08

**Authors:** Helga Agusta Sigurjonsdottir, Mikael Gronowitz, Ove Andersson, Robert Eggertsen, Hans Herlitz, Augustinas Sakinis, Bo Wangberg, Gudmundur Johannsson

**Affiliations:** 1 Department of Medicine, Centrum of Endocrinology and Metabolism, Sahlgrenska University Hospital, University of Gothenburg, Gothenburg, Sweden; 2 Nödinge Primary Health Care Center, Nödinge, Sweden; 3 Department of Medicine, Hypertension Outpatient Clinic, Sahlgrenska University Hospital, University of Gothenburg, Gothenburg, Sweden; 4 Department of Medicine Primary Health Care, Mölnlycke Primary Health Care and Research Centre, University of Gothenburg, Gothenburg, Sweden; 5 Department of Medicine, Nephrology Outpatient Clinic, Sahlgrenska University Hospital, University of Gothenburg, Gothenburg, Sweden; 6 Dept of Radiology, Sahlgrenska University Hospital, University of Gothenburg, Gothenburg, Sweden; 7 Dept of Surgery, Sahlgrenska University Hospital, University of Gothenburg, Gothenburg, Sweden

**Keywords:** Endocrine hypertension, Hyperaldosteronism, Aldosterone, Renin, Hypertension, Resistant hypertension, Adrenal hyperplasia

## Abstract

**Background:**

The existence of unilateral adrenal hyperplasia (AH) has been considered a rare cause of primary hyperaldosteronism (PA).

**Methods:**

In a prospective study we screened for PA in a non-selected (NSP) and selected hypertensive population (SP), to define the cause of PA. We included 353 consecutive patients with hypertension; age 20 to 88 years, 165 women and 188 men, from a university-based Hypertension and Nephrology Outpatient clinics (123 SP) and two primary care centres, (230 NSP) from the same catch-up area. Serum aldosterone and plasma renin activity (PRA) were measured and the ARR calculated. Verifying diagnostic procedure was performed in patients with both elevated aldosterone and ARR. Patients diagnosed with PA were invited for adrenal venous sampling (AVS) and offered laparoscopic adrenalectomy when AVS found the disease to be unilateral.

**Results:**

After screening, 46 patients, 13% of the whole population (22.8% SP and 7.8% NSP) had aldosterone and ARR above the locally defined cut-off limits (0.43 nmol/l and 1.28 respectively). After diagnostic verification, 20 patients (6%) had PA, (14.5% SP and 1.4% NSP). Imaging diagnostic procedures with CT-scans and scintigraphy were inconclusive. AVS, performed in 15 patients verified bilateral disease in 4 and unilateral in 10 patients. One AVS failed. After laparoscopic adrenalectomy, 4 patients were found to have adenoma and 5 unilateral AH. One patient denied operation.

**Conclusion:**

The prevalence of PA was in agreement with previous studies. The study finds unilateral PA common and unilateral AH as half of those cases. As may be suspected PA is found in much higher frequency in specialised hypertensive units compared to primary care centers. AVS was mandatory in diagnosis of unilateral PA.

## Background

Patients with primary hyperaldosteronism (PA) have been found to have increased rate of cardiovascular events compared to patients with primary HT [1]. PA is now considered the most common form of secondary HT. Studies from the last decade, using serum aldosterone/ renin ratio (ARR) for screening, have reported the prevalence of PA to be higher than previously thought i.e. from 4.6% to 22% in hypertensive populations [2,3], depending on whether it is a non-selected patient group (NSP) from primary care centres [2,4] versus selected patient group (SP) from specialised HT clinics [2-15], study design (prospective studies [2-7,9,11,13-15] versus retrospective [8,12,13]) and ethnicity of the studied population [2,3,6,12].

Detecting PA secures specified, target treatment and possible cure of the HT. Unilateral PA due to aldosterone producing adenoma (APA) or unilateral adrenal hyperplasia (AH) can be a curable cause of HT. APA has been diagnosed with imaging techniques and unilateral AH has been considered a rare form of PA [16,17].The biochemical cure rate of APA has been shown to increase to up to 100% with the use of adrenal vein sampling (AVS). AVS also minimizes the risk of adrenalectomy of a non-producing adrenal incidentaloma [18-20].

It is not clear which patients should be screened for PA [7,21,22] and there is no consensus on the cut-off limits for ARR as they are method dependent and influenced by environment and salt intake (salt-enriched or salt deprived). The proposed advantages of using ARR as a screening method is that this ratio is less influenced by anti-hypertensive medical treatment and the physiological variations of circulating levels of aldosterone and renin [21]. Using the ARR together with s-aldosterone as a screening tool has also been suggested to have higher specificity than using only ARR [13]. Using cut-off values above the 95^th^ percentile of both s-aldosterone and ARR in a normal population only 4.3% of screened hypertensive patients had false positive results for PA [7].

The aim of our study was screening for PA using locally produced cut-off limits of ARR and s-aldosterone, verify the PA with confirmatory testing and defining the aetiology by the use of computerised tomography, AVS and post-operative histopathology (PAD) when PA was found to be unilateral and surgically treated [22,23,25].

## Methods

### Subjects and methods used to find cut off limits for definition of PA

In order to define diagnostic limits for serum aldosterone levels and ARR we included 39 patients with well defined PA from the Sahlgrenska University Hospital and 36 healthy controls, all being Caucasian. A logistic regression model and a ROC curve were used to define the sensitivity and specificity of using s-aldosterone concentration and ARR as a diagnostic marker for PA. A subgroup analysis was performed using 16 patients (of the 39 with PA) who had histologically proven aldosterone producing adenoma (APA) post-operatively.

### Subjects and methods used for screening for PA

Screening for PA was performed in a SP (n = 230) at the Hypertension and Nephrology outpatient clinics at Sahlgrenska University Hospital, Gothenburg and in a NSP (n = 123) at two nearby primary care centres, Mölnlycke and Nödinge. Ninety percent of patients asked for participation, accepted, all being Caucasians, resulting in a total population of 353 patients (230 SP and 123 NSP). Inclusion criteria was HT defined as systolic and diastolic blood pressure (SBP and DBP respectively) above 140 mmHg and 90 mmHg respectively, for at least 3 months, measured on at least 3 different occasions, before antihypertensive treatment was initiated. Patients were included consecutively, having both treated and newly diagnosed HT. In the morning, after 10 minutes rest, blood pressure (BP) was measured twice with an accuracy of 2 mmHg using a mercury sphygmomanometer, with the patient in a sitting position. Blood samples for s-aldosterone and plasma renin activity (PRA) were then collected and ARR calculated. Urinary excretion of aldosterone and sodium was then measured from a 12 hour over-night urinary collection.

### Subjects and methods to verify PA

Patients found to have elevated ARR and s-aldosterone (group with suspected PA = SPA) were studied further in order to verify PA. At a follow-up visit, sampling of s-aldosterone and PRA was repeated after a 10-minute rest in a sitting position and the ARR calculated. The anti-hypertensive treatment was then changed in order to eliminate medications affecting the RAAS. If needed, patients received treatment with α-receptor inhibitor and/or potassium supplementation and Ca^2+^- antagonists. Four to six weeks later (six weeks when the patient had been treated with spironolactone), patients were studied again before and after a three days oral salt-loading test (10 g of NaCl daily in three doses) [23]. Normokalaemia was secured during those three days. An aldosterone urinary excretion rate of more than 28 nmol/day in the presence of urinary sodium excretion of more than 250 mmol/day on the last day of the salt-load is considered diagnostic for PA [21]. Patients having verified PA were studied further with CT of the adrenals, ^131I^-cholesterol-scintigraphy and adrenal vein sampling (AVS), in order to find the affected adrenal/adrenals.

### Methods for adrenal vein sampling (AVS) and unilateral Laparoscopic Adrenalectomy (LA)

An infusion, constituting 3 ml of cosyntropin (0.25 mg/ml) and 800 ml of 0.9% NaCl, was given (rate 100 ml/hour) for 90 minutes before the catheterisation or a total of 93.75 μg/hour of cosyntropin. Blood samples for cortisol and aldosterone were taken from the femoral vein, inferior vena cava and right and left adrenal veins (RAV and LAV). Samples from the RAV were analysed directly in order to secure a successful AVS. When the cortisol value from the RAV was at least 10 times higher than that from infra-renal IVC (inferior vena cava), the catheterisation could be completed after reaching the LAV (performed in the mean-time while the cortisol level from the RAV was being measured), otherwise, re-catheterisation of the RAV was done before concluding the AVS. Results from the AVS were then used to calculate the lateralisation ratio in order to quantify unilateral or bilateral overproduction of aldosterone [24,26-28]. The lateralisation ratio is found by calculating the ratio of aldosterone and cortisol for ipsilateral adrenal $\left(\text{IR}=\left({\text{Aldosterone}}_{\text{dominant adrenal vein}}/{\text{Cortisol}}_{\text{dominant adrenal vein}}\right)/\left({\text{Aldosterone}}_{\text{peripheralvein}}/{\text{Cortisol}}_{\text{peripheral vein}}\right)\right)$, for the contralateral adrenal $\left(\text{CR}=\left({\text{Aldosterone}}_{\text{non}-\text{dominant adrenal vein}}/{\text{Cortisol}}_{\text{non}-\text{dominant adrenal vein}}\right)/\left({\text{Aldosterone}}_{\text{peripheral vein}}/{\text{Cortisol}}_{\text{peripheral vein}}\right)\right)$ and then the lateral ratio IR/CR. In order to define unilateral disease; the IR should be >1.4, the CR <1 and the LR >3. When the lateralization ratio indicated a unilateral disease; the affected adrenal gland was removed by LA.

### Histopathological definition of adrenal hyperplasia vs. adenoma

The histopathological definition of AH and adenomas was in line with former descriptions [29,30]. AH would have histopathology corresponding to microscopic hyperplasia possible with nodular cortical hyperplasia and adenomas should have atrophied or otherwise normal cortex with a single adenoma.

### Analytical methods

The serum and urinary sodium and potassium were measured using ion selected electrode (Hitachi 911, Roche Diagnostics, GmbH, Mannheim, Germany). Radioimmunoassay (RIA) was used to measure serum and urinary aldosterone (Aldosteron MAIA, Adaltis Italia S.p.A., Roma, Italy).

The PRA determination involves an initial incubation of plasma to generate angiotensin I, followed by quantification of angiotensin I by RIA. The PRA was measured by one RIA (ABBOTT, Diagnostic Division, Kista-Stockholm, Sweden), for the first 227 patients. Due to the fact that Abbott discontinued marketing their RIA kit in the middle of our study, the last 126 patients had their PRA analyzed by another RIA method (DIA), Gammacoat R, DiasSorin Inc., Stillwater, USA. The latter method uses a procedure based on competitive binding principles of radioimmunoassay, where the antibody is immobilized onto the lower wall of the Gammacoat tube of the RIA kit. Seventy-four samples were run in parallel using both methods, and there was a highly significant correlation between the two measurements (r = 0.97, p <0.0001). The first 227 PRA values were corrected for the subsequent method in order to minimize the effect of changing methods. PRA had a within-run coefficient of variation of 8.8% and the lower detection limit was 0.15 ng/ml/h. Serum cortisol was measured by RIA (Orion Diagnostica, Orion Corporation Orion Diagnostica. Espoo, Finland).

### Ethics

Each participant received oral and written information of the study and signed an informed consent before being included into the study. The study was conducted according to the guidelines of the Helsinki Declaration of Helsinki and approved by the Regional Ethics Review Board at the University of Gothenburg.

### Statistical methods

To assess the diagnostic cut-off value of s-aldosterone and the ARR, the receiver operating characteristic (ROC) analysis was carried out.

A logistic regression model was performed to study the relation between the s-aldosterone level and risk of disease. In the analysis we plotted the ROC curve of s-aldosterone and of the ARR as a predictor for PA. From the analysis above, we then determined the best ‘cut-off’ for each measure of interest.

Wilcoxon test was used for within group comparison and for between-group comparisons the Mann–Whitney test was used for calculations. A p-value <0.05 was considered significant.

## Results

### The cut-off levels for s-aldosterone and ARR

In the analyses of the 39 patients with PA and 36 healthy controls, the best cut-off for s-aldosterone was 0.44 nmol/L (90% sensitivity and 91% specificity) and 1.28 (82% sensitivity and 83% specificity) for the ARR. In the analysis of the 16 patients with APA and the 36 healthy controls, the optimal cut-off for s-aldosterone was 0.43 nmol/L (94% sensitivity and 91% specificity) and 1.56 (94% sensitivity and 92% specificity) for ARR. The cut-off point for s-aldosterone and ARR used in the screening study was therefore decided to be 0.43 nmol/L for s-aldosterone and 1.28 for the ARR. In order to minimize false positive PA both limits were required.

### The screening phase

**Figure 1:**
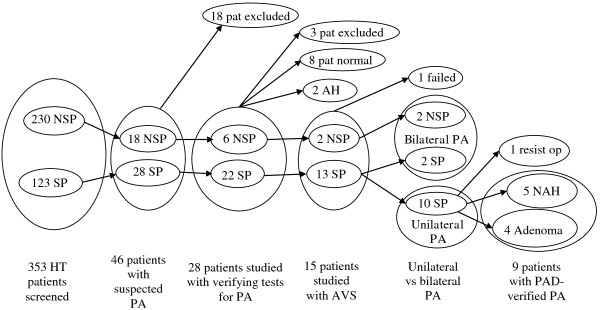
**The study populations.** From the whole screening population (n = 353), to the group found to have suspected primary hyperaldosteronism (n = 46) and the subsequent study group studied for the diagnose of PA all the way to PAD (post-operative histopathology) after endoscopic adrenal surgery. NSP = non-selective population, SP = selective population, pat = patients, NAH = nodular adrenal hyperplasia, PA = Primary aldosteronism, PAD = histopathological diagnosis

Three hundred fifty-three consecutive patients, age 20 to 88 years, were included, (Table 1, Figure 1). Seventeen patients were not on anti-hypertensive medication (Table 1). Patients with resistant hypertension, treated with more than 3 anti-hypertensive drugs were 9.4% of the whole group, 21% SP and 3.5% NSP.

**Table 1:** Description of study population and anti-hypertensive treatment of the groups

	**Population**					
	**The rest of the screening population**			**SPA-group**		
	**All (n = 307)**	**SP (n = 94)**	**NSP (n = 213)**	**All (n =46)**	**SP (n = 28)**	**NSP (n = 18)**
**Female**	164	47	117	18	11	7
**Male**	189	75	114	28	7	21
**Age (years)**	60.0 ± 11.1	56.0 ± 11.9	61.8 ± 10.3	59.7 ± 10.6	56.3 ± 10.8	65.1 ± 7.9
**BMI (kg/m**^**2**^**)**	28.7 ± 4.5	28.4 ± 5.2	28.8 ± 4.2	29.0 ± 4.2	28.6 ± 3.8	29.6 ± 4.7
**Mean SBP (mmHg)**	146.9 ± 21.0	149.9 ± 22.3	145.6 ± 20.4	152.0 ± 20.9	152.2 ± 20.9	152.7 ± 21.5
**Mean DBP(mmHg**	86.8 ± 10.6	87.5 ± 10.5	86.4 ± 10.7	91.0 ± 9.0	91.0 ± 8.1	90.9 ± 10.5
**Duration of HT (years)**	12.6 ± 9.8	13.1 ± 9.3	12.3 ± 10.1	12.9 ± 6.9	12.9 ± 5.6	12.8 ± 8.8
No of drugs*	1.8 ± 1.1	2.3 ± 1.3	1.6 ± 0.9	2.4 ± 1.1	2.6 ± 1.3	2.1 ± 0.8
**No of drugs 0 to 3**	279	75	204	39	21	18
**No of drugs 4 to 5**	26	18	8	7	7	0
**β-blockers**	165	60	105	34	22	12
**ACE – & AII-inhibitors**	154	60	94	24	16	8
**diuretics**	131	47	84	18	13	5
**Spironolactone**	0	0	0	2	2	0
**Potassium supplements**	14	8	6	3	3	0
**Serum Potassium (mmol/l)**	4.0 ± 0.4	3.9 ± 0.4	4.0 ± 0.4	3.7 ± 0.3	3.7 ± 0.5	3.7 ± 0.3

The rest of the screening population (n = 307 = 353-46, i.e. the whole screening population – the SPA group) and the group suspected with primary hyperaldosteronism (the SPA group) due to the defined cut-off limits for serum aldosterone and serum aldosterone/renin ratio (ARR). SP = selected population. NSP = non-selected population. No of medicine = total number of antihypertensive drugs at the time of screening, presented as the mean value ± standard deviation. Respective antihypertensive drug is presented as total number of patients treated with it. β-blockers = β –receptor inhibitor. ACE = angiotensin converting enzyme. AII = angiotensin II. *Information on drugs is missing for 2 patients not being in the SPA-group.

Forty-six patients (13%) had s-aldosterone and ARR above 0.43 nmol/L and 1.28, respectively, 28 SP (7.9%) and 18 NSP (5.1%). Thus 22.8% of the SP and 7.8% of the NSP were above the defined cut-off limits for PA at screening.

Comparing the 46 patients with suspected PA with the rest of the screening population (RP, n = 307) mean DPB was higher (p = 0.008, Table 1) and serum potassium lower (3.7 ± 0.3 mmol/L vs. 4.0 ± 0.4 mmol/L, p <0.0001). Also the group with suspected PA had more often a history of low serum potassium levels (14 of 46 vs. 26 of 307, p <0.0001). No difference was, however, found in body mass index (BMI), age, duration of HT or mean SBP (Table 1). Patients with suspected PA were more frequently treated with β-receptor blockers (74% vs. 54%, p <0.05), Ca2 + −channel blockers (54% vs. 35% p = 0.03) and peripheral vasodilators and α-receptor inhibitors, (59% vs. 36%, p = 0.01).

### Verification of PA

Of the 46 patients with suspected PA eighteen patients did not participate due to another concomitant illness, they had moved or refused further investigation. The 28 patients studied further are described in Table 2.

**Table 2:** Patients included in the study

	**Study number**	**Study popul-ation**	**ARR**	**Serum aldosterone (nmol/L)**	**CT**	^**131**^**I-chol-scint**	**AVS**	**Diagnose**	**PAD**
1	14	NSP	1.30	0.69	-	-	-	AH	
2	1042	NSP	2.50	0.54	+ (L)	-	-	AH	
3	1052	NSP	3.10	0.46	/	/	/	Normal	
4	1065	NSP	4.30	0.64	+ (R)	-	Excluded		
5	1092	NSP	1.50	0.75	/	/	/	Normal	
6	1105	NSP	2.00	0.62	/	/	/	Normal	
7	2002	SP	2.10	0.60	/	/	/	Normal	
8	2006	SP	3.80	0.49	- (x2)	+ (B)	+ (L)	APA (L)	NAH
9	2007	SP	4.30	0.64	+ (R)	-	+ (R)	APA (R)	NAH
10	2010	SP	2.90	0.43	-(?L)	-	+ (L?)	APA/AH	Adenoma
11	2014	SP	4.50	0.68	-	- (?R)	+ (R)	APA (R)	NAH
12	2022	SP	6.80	1.29	-	-	-	AH	
13	2036	SP	2.90	0.43	/	/	/	Normal	
14	2037	SP	4.50	0.95	/	/	/	Normal	
15	2042	SP	1.70	0.44	-	-	+ (L)	APA (L)	NAH
16	2049	SP	9.30	1.39	+	-	Excluded		
17	2060	SP	3.30	0.89	+ (R)	-	+ (R)	APA (R)	
18	2069	SP	1.64	0.56	/	/	/	Normal	
19	2070	SP	2.40	0.88	+ (L)	-	-	AH	
20	2074	SP	4.95	0.65	-	/	/	AH	
21	2082	SP	3.80	0.50	-	/	/	AH	
22	2083	SP	2.10	1.10	+ (R)	+ (R)	+ (R)	APA (R)	Adenoma
23	2084	SP	3.90	0.86	-	+ (L)	+ (L)	APA (L)	Adenoma
24	2086	SP	1.40	0.60	+ (L)	-	+ (L)	APA (L)	NAH
25	2099	SP	7.20	1.69	+ (L)	-	+ (L)	APA (L)	Adenoma
26	2100	SP	3.80	0.50	/	/	/	Normal	
27	3005	SP	2.10	0.62	-		Excluded		
28	3006	SP	3.50	0.94	-	-	failed		

Population defines if the patient was screened in a non-selective (NSP) or a selective (SP) hypertensive population. Values for serum aldosterone and the ratio of s-aldosterone/plasma renin activity (ARR) at inclusion and results of the adrenal CT (CT), ^131^I-norcholesterol-scintigraphy (^131^I-chol-scint) and adrenal vein sampling (AVS) are given. For the CT, ^131^I-chol-scint and AVS the following symbols are used +, - , / and failed, for positive, bilateral overproduction of aldosterone, not executed and failed investigation respectively. The letters (L), (R) and (B) indicate left, right and bilateral respectively. In the diagnose column, Normal, AH and APA are used for normal ie no hyperaldosteronism, adrenal hyperplasia and aldosterone producing adenoma respectively. In the PAD column, NAH is used for cortical nodular hyperplasia and adenoma for cortical adenoma.

After the adjustment of anti-hypertensive treatment, the mean value of s-aldosterone, PRA and ARR did not differ from those collected at the time of screening (data not shown). During drug adjustment two patients developed heart failure and were excluded from further evaluation and the third patient denied further evaluation at this point. Eight patients were found to have a normal salt-loading test (2.3% of the whole screening population and 29% of the 28 patients studied further) and were not studied further. Two of the patients found to be normal were treated with potassium supplements.

Of the 20 patients (5.7%) found to have PA after verification, there were 3 women and 17 men. Thus, 17 SP (14.5% of the SP) and 3 NSP (1.4% of the NSP) had verified PA, excluding the 18 drop outs from the original group.

Two of the 20 patients found to have PA, had results comparable to AH and a negative CT-adrenals (CT) (and then assumed to have bilateral PA at that time) and were not studied further (pat no 2074 and 2082 Table 2).

### Results of objective research, AVS, LA and histopathological findings

Nine patients had positive and 9 patients had negative findings on CT-adrenals (CT). Two patients had a visible adenoma in the left adrenal on CT although AVS demonstrated a bilateral disease. One patient had only a vague positive finding on CT, unilateral overproduction of aldosterone by AVS and after LA, PAD confirmed APA. One patient had a negative CT finding, unilateral overproduction of aldosterone by AVS and PAD after LA confirmed APA. Three patients had positive findings on 131I-cholesterol-scintigraphy and 14 negative, six of those 14 patients were found to have unilateral overproduction of aldosterone by AVS. One patient with bilateral positive finding on scintigraphy had unilateral overproduction of aldosterone after AVS and PAD confirmed unilateral AH (See Table 2).

Fifteen patients were studied with AVS and 3 patients were excluded (two denied to be studied further and one was excluded due to age). Four patients had lateralisation ratio after AVS indicating bilateral AH and received medical treatment. Ten patients were found to have unilateral PA and 9 of them went further to LA, one patient denied operation.AVS failed to reach the RAV in one patient.

After LA, five of the 9 patients were diagnosed with AH and 4 with adenoma, verified by PAD (Table 2). Histopathological findings found that all adenomas demonstrated otherwise normal or atrophied adrenal cortical structure except one that also has nodular hyperplasia. All specimens diagnosed with hyperplasia had a microscopic character of nodular cortical hyperplasia.

### Results on BP and serum potassium after LA

Three months after LA, five of the nine patients, had fewer anti-hypertensive drugs, BP was somewhat lower in three other patients with the same anti-hypertensive treatment as before the LA and one patient had 5 anti-hypertensive drugs after LA compared to three before (Table 3).

**Table 3:** Results on blood pressure, anti-hypertensive drugs and potassium supplementation in patients treated with Laparoscopic Adrenalectomy

**Study number**	**At baseline**			**Laparo-scopic Adrenal-ectomy**	**3 months after Laparoscopic Adrenalectomy**			**1 year after Laparoscopic Adrenalectomy**		
		**BP (mmHg)**	**No anti-HT drugs**	**K + subst. yes/no**	**PAD**	**BP (mmHg)**	**No anti-HT drugs**	**K + subst. yes/no**	**BP (mmHg)**	**No anti-HT drugs**	**K + suppl. yes/no**
2006	162/100	4	no	NAH	154/90	2	no	128/80	3	no
2007	170/95	2	no	NAH	140/100	2	no	120/82	2	no
2010	185/107	3	yes	Adenoma	154/94	3	yes	215/110	3	yes
2014	164/97	4	no	NAH	165/85	3	no	-	-	-
2042	167/94	3	no	NAH	130/88	5	no	-	-	-
2083	150/90	2	yes	Adenoma	135/75	1	no	150/70	-	no
2084	141/78	1	no	Adenoma	120/70	1	no	-	-	-
2086	158/89	4	yes	NAH	140/86	3	no	-	-	-
2099	138/94	3	no	Adenoma	-	2	-	140/80	0	no

Results are presented for data at baseline, three months after Laparoscopic Adrenalectomy and one year after Laparoscopic Adrenalectomy. Blood pressure (BP), number of Anti-hypertensive drugs (No anti-HT drugs), potassium substitution (K + subst.), post-operative histopathology (PAD), Nodular adrenal hyperplasia (NAH).

One year after LA, two patients are treated with fewer anti-hypertensive drugs, one of them being cured from HT and not needing anti-hypertensive drugs at all. Two other patients were treated with the same amount of anti-hypertensive drugs after LA as before. Further information on anti-hypertensive drugs used one year after LA was missing for 5 patients (Table 3).

All the nine patients had serum potassium levels in the upper normal reference values at 3 and 12 months after LA. Two of the three patients needing potassium substitution before LA were cured by LA, in one of them information on potassium substitution one year after LA was missing but 3 months after LA the need for potassium substitution was cured (Table 3).

## Discussion

In summary, after prospective screening of a hypertensive population, unilateral AH was found to be as common as APA in patients with PA and unilateral disease.

Earlier publications suggest that unilateral AH is an uncommon cause of unilateral PA with the highest reported prevalence being 1 of 3 operated cases [31]. Our study indicates that the prevalence of unilateral AH is higher. In a recent review on the subject unilateral PA is presented as 2% of PA, bilateral hyperplasia as 60% and unilateral adenoma as 35% [32]. Our results are in huge discrepancy with this. By using AVS and lateralization index a unilateral dominant disease can be defined although it does not preclude the possibility of the contra lateral gland being entirely free from hyperplasia [33]. As adrenalectomy with the use of a laparoscopic technique has become much easier and to be cost-beneficial over long-term medical therapy, it is of increasing interest to find patients with unilateral PA eligible for surgical therapy [34]. Surgical removal of APA or unilateral AH has been found to normalise hypokalaemia in all cases, improve HT in 60-90% of the cases, reverse left ventricular geometry and alter myocardial texture [35] and not the least increase quality of life [36].

Our results support that patients with verified PA should be further studied with AVS in order to determine whether a unilateral or a bilateral disease is at hand, as previously suggested [15,37]. It has been postulated that surgically correctable PA in the HT population is less than 1.5%, in this study we found it to be 2.6% and as high as 59% of those with confirmed PA (10 of 17 patients) [38]. The results on BP, anti-hypertensive treatment and need for potassium substitution before, 3 months and one year after surgery lend further support to this conclusion (Table 3).

We found positive screening results in a high proportion of patients with HT in both the specialised (22.8%) and non-specialist (7.8%) hypertensive units, which is, in line with recent studies [2-15].Our study therefore confirms previous conclusions that awareness of PA should be high among patients with HT in both SP and NSP [33,39].

The importance of screening is signified by the fact that patients with PA have more HT related organic complications compared to those with primary HT [1,40-47]. Which patients should be screened? The clinical features of patients with PA are not specific as they can have normal potassium concentrations and be without treatment resistant HT [21,25]. Even so, our study indicates that patients with a resistant increase in DBP and in the lower normal interval of serum potassium should be included in the population investigated for PA.

The caveats of using CT in detection of APA has to do with the smallness of the adenomas that can easily be missed as well as the fact that many adrenal adenoma are non-functioning [48]. We found imaging results to be confounding in up to half of the cases and the use of scintigraphy was not helpful in the etiological work-up of patients with PA. This emphasizes the importance of using AVS in detection of unilateral vs. bilateral PA. AVS needs to be executed by an experienced radiologist, due to the anatomy of the right adrenal vein, that often causes a failing procedure. This indicates that AVS should only be performed in specialised centers.

The limitations of our study are the size of the study population, the numbers of drop outs restraining the final confirmation test. Moreover, bilateral AH cannot be totally excluded as only the adrenal gland with the dominant production of aldosterone was surgically removed in those cases classified an unilateral AH. The strength of our study is, however, the locally defined cut-off limits for ARR and s-aldosterone used for screening. The study was also prospective in consecutive patients from both NSP and SP from the same catch up area.

## Conclusion

We conclude that in this prospective study the overall prevalence of PA is higher among patients with HT cared for in a specialised unit. The use of AVS was found to be mandatory in the detection of unilateral PA as imaging results and scintigraphy, were unreliable in that perspective. In this study, unilateral AH was as common as unilateral APA.

## Competing interests

The authors declare that they have no competing interest.

## Authors’ contributions

HAS, MG, OA, RE, HH and GJ all made substantial contribution in the conception and study design, execution of the study, analysis and interpretion of data. AS executed all adrenal vein samplings and contributed with designing that part of the study. BW executed all laparoscopic adrenalectomies. AS and BW also contributed in interpretation of data. All authors have been involved in drafting the manuscript and revising it critically and read and approved the final manuscript.

## Funding

This work was supported by the Health & Medical Care Committee of the Regional Executive Board, Region Västra Götaland, Sweden.

## Pre-publication history

The pre-publication history for this paper can be accessed here:

http://www.biomedcentral.com/1472-6823/12/17/prepub
